# Clinical, Epidemiological, and Dermoscopic Features of Different Clinical Variants of Cutaneous Lichen Planus in the Indian Population: A Prospective Observational Study

**DOI:** 10.7759/cureus.86051

**Published:** 2025-06-15

**Authors:** Ashna Malhotra, Arvind Krishna, Robin Chugh, Abhinav David

**Affiliations:** 1 Department of Dermatology, Venereology and Leprosy, Subharti Medical College, Meerut, IND

**Keywords:** dermoscopy, histopathology, lichen planus, pigmentation, wickham striae

## Abstract

Introduction: Lichen planus (LP) is a distinct clinical entity that commonly affects the skin, hair, nails, and mucous membranes. Multiple clinical variants of LP with varying morphologies have been documented. This study aimed to analyze the clinical, epidemiological, and dermoscopic features of different variants of cutaneous LP in the Indian population.

Methods: This prospective observational study was conducted at a tertiary care hospital from January 2021 to August 2022. A total of 50 patients with a confirmed diagnosis of cutaneous LP were included. Demographic details, including age, disease duration, symptoms, and family history, were recorded.

Results: Among the 50 patients, 30 (60%) were female and 20 (40%) male, with an age range of 8-65 years (mean age: 37 ± 14 years). Classical LP was the most common variant observed (26 cases, 52%), followed by lichen planus hypertrophicus (LPH) in eight cases (16%) and eruptive LP in six cases (12%). Wickham’s striae (WS) was the most common dermoscopic finding in classical LP (observed in 23 cases, 88.5%), but was absent in LPH, actinic LP, and lichen planus pigmentosus (LPP). The most frequent pigmentation patterns in classical LP were diffuse dots and diffuse globules, seen in three cases (11.5%). Pigmentation patterns were more variable in LPH and actinic LP. The background color was pink in classical and eruptive LP, and brownish in LPH, actinic LP, and LPP. It showed mixed shades in annular atrophic LP. No significant differences in demographic parameters were noted among the different variants.

Conclusion: WS was the most consistent dermoscopic feature in cases of classical cutaneous LP and can assist physicians in diagnosis. Detailed dermoscopic evaluation, combined with a comprehensive clinical history, is essential for diagnosing classical and atypical LP variants and differentiating them from clinically similar conditions. This approach offers important clues regarding the underlying histopathology.

## Introduction

Lichen planus (LP) is a chronic inflammatory mucocutaneous condition with diverse morphological and histopathological features. Classically, LP presents as pruritic lesions and commonly affects the skin, hair, nails, and mucous membranes. The global prevalence of LP in the general population and among dermatology outpatients is approximately 0.89% and 0.98%, respectively [1]. In India, based on limited studies, the reported prevalence ranges from 0.5% to 77% [2-4].

LP manifests in various forms based on morphological and etiological characteristics, with cutaneous LP and oral LP being the most common variants [5,6]. Etiological factors include autoimmune mechanisms and genetic predisposition. T-lymphocyte-mediated cytotoxic activity plays a central role in the pathogenesis of LP [7]. A few studies have also reported genetic susceptibility to LP, including associations with specific human leukocyte antigen (HLA) allele class I (HLA B7) [8,9] and class II (HLA DR1 and DR12) [10,11], indicating a potential genetic component [12,13].

Despite advancements, further clinical and immunological research is needed to better understand the cytological characteristics of LP variants. Dermoscopy, a non-invasive diagnostic tool, serves as a bridge between microscopic dermopathology and clinical dermatology by allowing visualization of skin features not visible to the naked eye [14]. It plays a pivotal role in LP diagnosis, especially in cases where certain variants are clinically inaccessible or difficult to identify [15].

Dermoscopy has improved differentiation between LP and other dermatoses, such as psoriasis, with high diagnostic accuracy [16]. Common dermoscopic findings in LP include Wickham’s striae (WS), red dots or lobules (vascular structures), and deeper brownish or dotted pigmentation patterns (suggestive of hyperpigmentation) [14]. Although interface dermatitis forms the histopathological basis of LP, not all classical features are present in every case [17]. Furthermore, LP presents with several uncommon or atypical forms that pose diagnostic challenges. Therefore, it is essential to assess the clinical, epidemiological, and dermoscopic characteristics of LP variants to facilitate early and accurate diagnosis and prevent pigmentary sequelae.

This study aimed to analyze the clinical, epidemiological, and dermoscopic features of different variants of cutaneous LP.

## Materials and methods

This was a prospective observational study conducted between January 2021 and August 2022 in the Department of Dermatology, Venereology and Leprosy, Chhatrapati Shivaji Subharti Hospital, Meerut, India. The University Ethics Committee (Medical), Swami Vivekanand Subharti University, approved the study (approval number: SMC/UECM/2021/181, dated January 25, 2021). All participants provided written informed consent after receiving a full explanation of the study's purpose and procedures.

Eligibility criteria

Patients aged 18 years and above, clinically diagnosed with cutaneous lichen planus based on clinical features, and willing to provide written informed consent were included in the study. Patients with mucosal or nail LP were excluded.

Sample size calculation

The sample size for this observational study was estimated based on the formula for estimating a proportion with a specified relative precision: $n=\frac{Z^{2}&times;P&times;Q}{&delta;^{2}}$, where Z=1.96 for 95% confidence interval, P=0.03 (3% prevalence of LP in the Indian subcontinent), Q=1−P=0.97, δ=0.05 (margin of error).

This calculation yielded n = 44.72, which was rounded up to a final sample size of 50 patients. The value of p = 0.03 was derived from literature reporting that the prevalence of lichen planus varies from 0.1% to 3%, with higher rates observed in the Indian population [18]. A total of 50 patients with clinically diagnosed cutaneous lichen planus were thus screened and included.

Data collection

Detailed medical histories were recorded, and a comprehensive general physical examination, including vital signs, was performed. Dermoscopy was conducted using a commercially available dermoscope (Dino-Lite 2.0 USB; AnMo Electronics Corporation, Hsinchu City, Taiwan) for all participants. Findings were documented in a standardized format.

Statistical analysis

Continuous variables were summarized as mean ± standard deviation (SD), while categorical variables were expressed as frequencies and percentages. Comparisons between classical and atypical forms of LP were made using appropriate statistical tests. Student’s t-test was used for continuous variables, and the Chi-square test was applied for categorical variables. A p-value < 0.05 was considered statistically significant. Statistical analysis was performed using IBM SPSS Statistics for Windows, version 22 (Released 2013; IBM Corp., Armonk, New York, United States).

## Results

Demographics

Out of 50 patients, 30 (60%) were female and 20 (40%) were male with a ratio of 3:2. The frequency of LP was highest among patients aged 20-40 years, accounting for 26 (52%), followed by the age group of 40-60 years (n=18; 36%), <20 years (n=5; 10%), and >60 years (n=1; 2%). The mean age of patients included in the study was 37±14 years (age range: 8-65 years). The demographic characteristics of patients are given in Table 1.

**Table 1:** Demographic characteristics of participants (N=50) LP: lichen planus

Variables	Number of patients	Percentage
Age group (in years)		
<20	5	10%
20-40	26	52%
40-60	18	36%
>60	1	2%
Sex		
Male	20	40%
Female	30	60%
LP variant distribution		
Classical LP	26	52%
LP hypertrophicus	8	16%
Eruptive LP	6	12%
Actinic LP	4	8%
LP pigmentosus	3	6%
Annual atrophic LP	2	4%
Linear LP	1	2%
Duration of symptoms (in years)		
0-1	35	70%
1-2	8	16%
2-3	3	6%
>3	4	8%
Family history		
Yes	6	12%
No	44	88%

The mean duration between the onset of symptoms and presentation was 15.0±14.9 months (range: three weeks to five years). A duration of ≤1 year was observed in 35 (70%) patients, followed by one to two years in eight (16%), two to three years in three (6%), and >3 years in four (8%). A prior family history of LP was reported in six (12%) patients. Lesions were most commonly located on the extremities in 25 (50%) patients, followed by mixed distribution in 18 (36%) and central distribution in seven (14%). The initial site of lesion appearance was the limbs in 42 (84%) patients, the face in six (12%), and the trunk in two (4%). The Koebner phenomenon was observed in 26 (52%) patients.

Classical LP vs. atypical LP

Among the clinical variants of cutaneous LP encountered, the most common form of LP was classical LP, observed in 26 (52%) patients. This was followed by LP hypertrophicus (LPH) in eight (16%) and eruptive LP in six (12%) patients. Actinic LP, LP pigmentosus (LPP), and annual atrophic LP were seen in four (8%), three (6%), and two (4%) patients, respectively. A single case of linear LP was reported in one (2%) patient (Table 1). Overall, 34 (68%) patients experienced severe itching, while 9 (18%) had moderate itching and 7 (14%) mild itching. All patients with LPH reported severe itching. A history of topical application was present in 20 (40%) patients. Baseline characteristics were compared between the two groups. There was no statistically significant difference in age, gender, family history, lesion distribution, itching, and topical application between the two groups (Table 2).

**Table 2:** Baseline characteristics of classical vs. atypical LP LP: lichen planus

Variables	Classic LP (n=26)	Atypical LP (n=24)	P-value
Age (in years), mean±SD	37.1±13.8	35.9±14.1	0.762
Gender, n (%)			0.817
Male	10 (38.5%)	10 (41.7%)	
Female	16 (61.5%)	14 (58.3%)	
Family History, n (%)	3 (11.5%)	3 (12.5%)	>0.999
Mean duration (in months), mean±SD	13.8±10.9	16.3±18.7	0.576
Lesion distribution, n (%)			0.192
Central	2 (7.7%)	5 (20.8%)	
Extremity	16 (61.5%)	9 (37.5%)	
Mixed	8 (30.8%)	10 (41.7%)	
Itching, n (%)			0.361
Mild	2 (7.7%)	5 (20.8%)	
Moderate	6 (23.1%)	3 (12.5%)	
Severe	18 (69.2%)	16 (66.7%)	
Topical Application, n (%)			0.399
Yes	12 (46.2%)	8 (33.3%)	
No	14 (53.8%)	16 (66.7%)	

Clinical and dermoscopic findings

**Figure 1:**
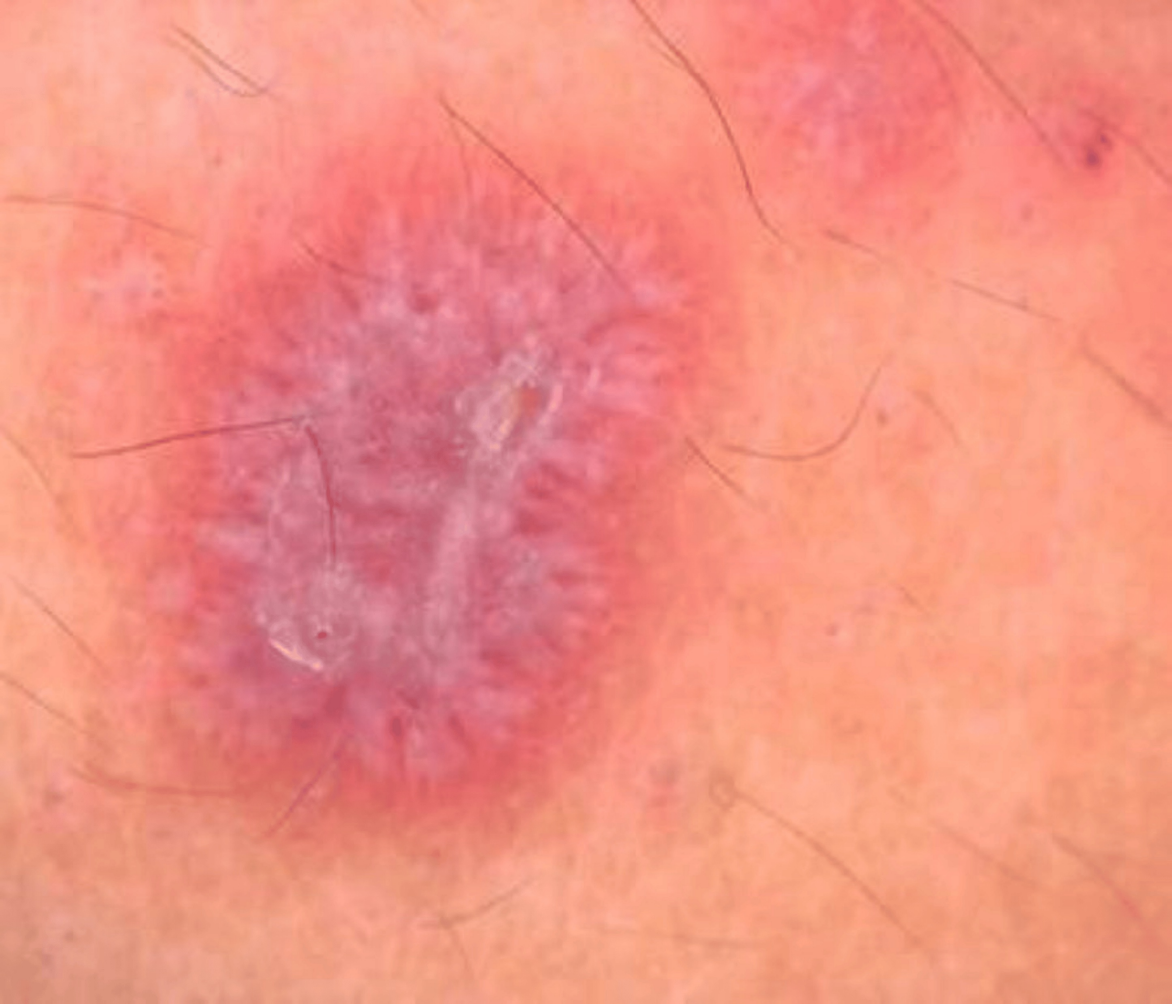
Dermoscopic findings in classical lichen planus, pink background, white radial streaming Wickhams striae, red dots

**Figure 2:**
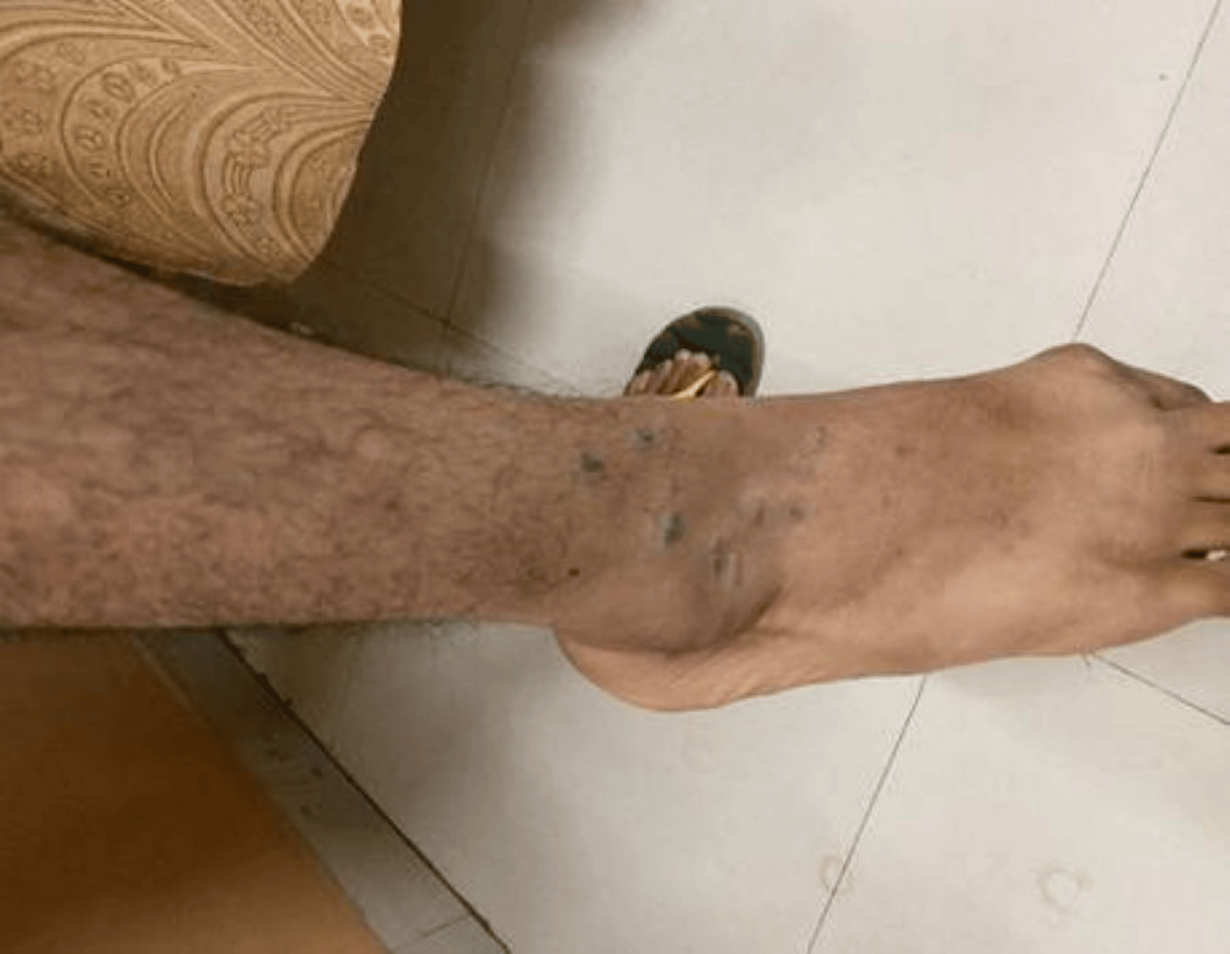
Clinical photograph of classical lichen planus in a 22-year-old male participant who presented with multiple violaceous papules and plaques over bilateral ankles, associated with severe pruritus

Dermoscopic findings corresponding to different variants of cutaneous LP (classical and atypical) were presented. Among the 26 patients with classical LP, WS was observed in 23 (88.5%) cases and was absent in three (11.5%). The most common pattern of WS was the white reticular pattern, seen in 13 (50%) patients, followed by a white veil-like pattern in four (15.4%) patients. Pigmentary patterns were observed as diffuse dots in three (11.5%) and diffuse globules in three (11.5%) patients, while 14 (53.8%) patients showed no pigmentary findings. A pink background color was the most common background finding, noted in 18 (69.2%) patients. Vascular patterns in the form of red dots were seen in 15 (57.7%) patients, and diffuse scaling was observed in seven (26.9%). Dermoscopic patterns in patients with classic LP are shown in Figures 1, 2, and summarized in Table 3.

**Table 3:** Dermoscopic patterns in classical LP (N =26)

Feature	Frequency (Percentage)
Wickham Striae	
Absent	3 (11.5%)
White veil like	4 (15.4%)
White (yellow) circular	1 (3.8%)
White linear	2 (7.7%)
White radial streaming	3 (11.5%)
White reticular	13 (50%)
Pigment Pattern	
Absent	14 (53.8%)
Cobblestone, yellow dots	1 (3.8%)
Diffuse dots	3 (11.5%)
Diffuse globules	3 (11.5%)
Diffuse homogenous	1 (3.8%)
Diffuse peppering	1 (3.8%)
Homogenous brown	1 (3.8%)
Reticular	2 (7.7%)
Vascular Pattern	
Absent	8 (30.8%)
Linear	3 (11.5%)
Red dots	15 (57.7%)
Background Color	
Brown	3 (11.5%)
Pink	18 (69.2%)
Violet	5 (19.2%)
Scales	
Absent	11 (42.3%)
Diffuse	7 (26.9%)
Patchy	3 (11.5%)
Peripheral	5 (19.2%)

The dermoscopic findings of different clinical variants of LP are summarized in Table 4. In LPH, WS was absent in all patients. Pigmentary patterns were variable, vascular patterns were absent in seven out of eight cases, the background color was brown in seven patients, and patchy scales were noted in six patients. For eruptive LP, WS appeared as either white radial or white reticular patterns (three patients each). Pigmentary patterns were either absent or showed diffuse homogeneous pigmentation (three patients each). Vascular patterns were absent in all cases. A pink background was noted in four out of six patients, and scales were absent in all. In actinic LP, WS was absent in all patients. Pigmentary patterns were variable, vascular patterns were absent in three out of four cases, the background color was brown in all cases, and scales were absent in three out of four patients. In LPP, WS was absent. Pigmentary pattern showed a perifollicular annular appearance in two out of three cases (66.7%). Vascular patterns were absent in two (66.7%) cases. The background color was brown in all cases, and scales were absent. In annular LP, WS pigmentary pattern showed diffuse peppering, vascular pattern was linear, background color was variable, and scales were absent. In the single case of linear LP, WS appeared as a white veil-like pattern. The pigment was yellow with a cobblestone appearance, the vascular pattern was absent, the background color was violet, and the scales were patchy.

**Table 4:** Dermoscopic patterns seen in various clinical variants of LP LP: lichen planus; LPH: lichen planus hypertrophicus; LPP: ichen planus pigmentosus

Feature	Classical LP (n=26)	LPH (n=8)	Eruptive LP (n=6)	Actinic LP (n=4)	LPP (n=3)	Annular atrophic LP (n=2)
Wickham Striae	Variable	Absent (8/8)	White radial/reticular (6/6)	Absent (4/4)	Absent (3/3)	Variable
Pigment Pattern	Variable	Variable	Diffuse homeogenous/absent (6/6)	Variable	perifollicular annular (2/3)	Diffuse peppering (2/2)
Vascular Pattern	Variable	Absent (7/8)	Absent (5/6)	Absent (3/4)	Absent (2/3)	Linear (2/2)
Background Color	Variable	Brown (7/8)	Pink (4/6)	Brown (4/4)	Brown (3/3)	Variable
Scales	Variable	Patchy (6/8)	Absent (6/6)	Absent (3/4)	Absent (3/3)	Absent (2/2)

## Discussion

The present study brings out in detail the clinical, epidemiological, and dermoscopic characteristics of patients with LP, a chronic inflammatory disease with worldwide prevalence of 0.9-2.2% [19,20]. In comparison, a study by Bhattacharya et al. reported an overall prevalence of 0.38% among patients attending a tertiary care centre in North India [21]. 

The term "Lichen Planus" originates from the Greek word "Liechen" meaning "tree moss" and "Planus" meaning "flat", referring to the surface morphology of the lesions. Clinically, LP is characterized by involvement of the skin, nails, hair, and/or mucous membranes [22]. A cell-mediated immune response, predominantly involving CD8+ T cells, plays a central role in the pathogenesis of LP and constitutes the main infiltrate observed within the epidermis [23]. While subtle clinical signs may guide diagnosis, dermoscopy provides a valuable, non-invasive tool to visualize underlying histopathological features. In this study, dermoscopic features assessed included WS (vein-like, circular, linear, radial streaming, reticular), vascular patterns (linear/red dots), scales (diffuse/patchy/peripheral), and pigmentation patterns (cobblestone dots, diffuse dots, diffuse globules, diffuse homogeneous, diffuse peppering, homogeneous brown, reticular).

In this study, the majority of LP patients were aged 20-40 years (n=26, 52%), followed by those aged 40-60 years (n=18, 36%), <20 years (n=5, 10%), and >60 years (n=1, 2%). This age distribution was similar to that reported by Anbar et al. [24], where most patients fell within the 21-50 age group, and by Bhattacharya et al. [21], where the majority were aged 20-49 years.

The overall duration of LP symptoms ranged from three weeks to five years, with 70% of patients presenting within 12 months of symptom onset. This finding aligns with studies by Singh et al. [25] and Bhattacharya et al. [21], in which 85% and 74% of LP patients, respectively, had a disease duration of one to six months. The female-to-male ratio was 3:2 (40% males, 60% females), similar to the 3:2 ratio reported by Singh et al. [25].

In our study, classical LP accounted for the majority of LP cases (52%), followed by LPH (16%), eruptive LP (12%), actinic LP (8%), LPP (6%), and annular atrophic LP (4%). Classical LP was found to be the most common LP subtype, consistent with findings from previous studies, including Bhattacharya et.al (47.4%) [21] and Garg et.al (30%) [26]. The most affected age group for classical LP was 20-40 years.

Among classical LP cases, the most frequently observed dermoscopic feature was pink pigmentation (69.2%), followed by red dots (vascular pattern) in 57.7%, and diffuse scales in 26.9%. Dermoscopic features were found to be highly variable. Red dots were the most common vascular pattern seen in classical LP patients (57.7%), with a few cases in eruptive LP (n=1, 16.7%), actinic LP (n=1, 25%), and LPH (n=1, 12.5%). On the other hand, Vázquez-Lopez et al. reported red vascular lines in 12% and red dots in 80% of cases [27], while Güngör et al. found red dots in 15.9% of cases [28]. Linear vascular patterns were predominantly seen in annular atrophic LP (n=2, 100%) and in one case of LPP (n=1, 33.3%).

White reticular WS was present in 50% of classical LP cases. WS is a hallmark dermoscopic feature of LP [29], associated histologically with compact orthokeratosis over zones of wedge-shaped hypergranulosis, particularly around acrotrichia and acrosyringia [30]. Dermoscopy, therefore, serves as a non-invasive, cost-effective method to identify WS in LP. 

WS was also prominently observed in eruptive LP and has been previously reported by Vázquez-López et al. using dermoscopy [27]. In classical LP patients, specific patterns of WS included white vein-like (n=4, 15.4%), circular (n=1, 3.8%), and linear (n=2, 7.7%) configurations. In annular atrophic LP, linear WS were present in 50% of cases (n=1). Eruptive LP exhibited white streaming WS (n=3, 50%) and white reticular WS (n=3, 50%). Overall, WS was observed in 26.9% of classical LP patients in this study, which is lower compared to findings by Güngör et al. (89.4%) [28] and Garg et al. (93.3%) [26]. WS was absent in all patients with LPH, actinic LP, and LPP.

Patchy scale patterns were observed most commonly in LPH patients (n=6, 75%), followed by classical LP patients (n=3, 11.5%) and actinic LP patients (n=1, 25%). No patchy scales were seen in patients with eruptive LP, LPP, or annular atrophic LP. Diffuse scales were more frequently noted in classical LP (n=7, 26.9%) and LPH (n=2, 25%) patients. Overall, 20% of LP patients exhibited patchy scales, while 18% showed diffuse scales. Lesions located in occlusive or less exposed areas may lack scaling due to poorly defined lichenification and lesion margins [30].

Cobblestone pigmentation (yellow dots) was observed in 12% of all LP patients, including one classical LP patient (3.8%) and 5 LPH patients (62.5%). Diffuse peppering pigmentation was noted in 10% of the total LP cases, including one classical LP patient (3.8%), two actinic LP patients (50%), and two annular atrophic LP patients (100%). Garg et al. reported diffuse peppered pigmentation in 28.57% of classical LP patients [26]. Diffuse globules were identified in six LP patients (12%), comprising three classical LP patients (11.5%), two LPH patients (25%), and one actinic LP patient (25%). In comparison, a cross-sectional study by Tatawati et al. reported brown globules in 40% of classical LP patients alone [3].

Perifollicular annular pigmentation was seen in 66.7% of LPP patients (two out of three), consistent with findings from Friedman et al. (53%) [14] and Güngör et al. (53.3%) [28]. The peppered pigmentation pattern was a common feature among classical LP patients in our study and aligns with observations reported in other studies [21,26,27,31].

Limitation

One limitation of our study is the relatively small sample size, which may restrict the generalizability of the findings. A larger sample size would enhance the robustness of the conclusions and provide a more comprehensive understanding of the clinical, epidemiological, and dermoscopic features of LP. Nevertheless, this study contributes valuable insights that can support further research and clinical advancements in the diagnosis and management of LP.

## Conclusions

This study highlights the consistency of clinical, epidemiological, and dermoscopic patterns across various clinical variants of LP. Dermoscopy, in conjunction with clinical and epidemiological evaluation, facilitates accurate visualization and documentation of WS, pigmentary changes, and vascular features in LP. The presence of red dots (vascular structures), pigmentation, WS, and scaling, well-documented in several observational studies, was also prominently observed in our study. WS emerged as a common dermoscopic feature in cutaneous LP, aiding in clinical diagnosis. Careful dermoscopic examination, combined with a thorough patient history, is essential for diagnosing both classical and atypical forms of LP and for differentiating it from clinically similar dermatoses. This approach provides critical insights into the underlying histopathology. 
